# Mutations changing tropomodulin affinity for tropomyosin alter neurite formation and extension

**DOI:** 10.7717/peerj.7

**Published:** 2013-02-12

**Authors:** Natalia Moroz, Laurent Guillaud, Brinda Desai, Alla S. Kostyukova

**Affiliations:** 1 Voiland School of Chemical Engineering and Bioengineering, Washington State University, Pullman, WA, USA; 2 Cellular and Molecular Synaptic Function Unit, Okinawa Institute of Science and Technology - Graduate University, Kunigami, Okinawa, Japan; 3 Department of Neuroscience and Cell Biology, Robert Wood Johnson Medical School, Piscataway, NJ, USA

**Keywords:** Tropomodulin, Tropomyosin, Actin, Neurite formation, Cytoskeleton

## Abstract

Assembly of the actin cytoskeleton is an important part of formation of neurites in developing neurons. Tropomodulin, a tropomyosin-dependent capping protein for the pointed end of the actin filament, is one of the key players in this process. Tropomodulin binds tropomyosin in two binding sites. Tmod1 and Tmod2, tropomodulin isoforms found in neurons, were overexpressed in PC12 cells, a model system for neuronal differentiation. Tmod1 did not affect neuronal differentiation; while cells expressing Tmod2 showed a significant reduction in the number and the length of neurites. Both tropomodulins bind short α-, γ- and δ-tropomyosin isoforms. Mutations in one of the tropomyosin-binding sites of Tmod1, which increased its affinity to short γ- and δ-tropomyosin isoforms, caused a decrease in binding short α-tropomyosin isoforms along with a 2-fold decrease in the length of neurites. Our data demonstrate that Tmod1 is involved in neuronal differentiation for proper neurite formation and outgrowth, and that Tmod2 inhibits these processes. The mutations in the tropomyosin-binding site of Tmod1 impair neurite outgrowth, suggesting that the integrity of this binding site is critical for the proper function of Tmod1 during neuronal differentiation.

## Introduction

Assembly and re-assembly of the actin cytoskeleton is an important part of neuritogenesis (for reviews see, da Silva & Dotti, 2002; Stiess & Bradke, 2011). The ability of actin to polymerize and depolymerize allows postmitotic neurons to migrate, and neurites, neuronal appendages, to sprout. Proteins that control polymerization at the barbed (fast-growing) ends and depolymerization at the pointed (slowly-growing) ends of actin filaments are of great importance for cytoskeletal reorganization.

Tropomodulin (Tmod), a capping protein for the pointed end (Weber et al., 1994), is one of the key players in this system. So far, little attention has been paid to Tmod function in neurons, mostly because it was believed that the most important things happen at the barbed end, where continuous incorporation of actin subunits occurs. Barbed ends face and push the distal membrane in growing neurites. The pointed end was labeled only as the end where depolymerization occurred; capping of this end was not believed to be crucial for neuritogenesis. However, recently it has been shown that Tmod plays an important role as a negative regulator in neurite formation (Fath et al., 2011).

There are four known Tmod isoforms: Tmod1 is found mainly in erythrocytes, but also in heart, skeletal muscle, brain, and many other tissues; Tmod2 is found in brain; Tmod3 is expressed widely in various tissues; Tmod4 is found in skeletal muscle only (Watakabe, Kobayashi & Helfman, 1996; Almenar-Queralt et al., 1999; Cox & Zoghbi, 2000; Conley et al., 2001). Hyperactivity, reduced sensorimotor gating, and impaired learning and memory were found in mice lacking Tmod2 (Cox et al., 2003). Tmod1 levels in Tmod2 knockout mice increased drastically, probably to compensate for the absence of Tmod2, while no change in the Tmod3 level was detected. In N2a neuroblastoma cells (model system to study neuritogenesis), both Tmod1 and Tmod2 were expressed, but levels of Tmod1 increased drastically starting 24 hours after induction of neuritogenesis (Fath et al., 2011).

The Tmod molecule consists of two functionally and structurally distinct halves, a disordered N-terminal domain, and a compact C-terminal domain (Kostyukova et al., 2000; Krieger et al., 2002). In order to bind tightly to actin, Tmod requires tropomyosin (Weber et al., 1994). There are two tropomyosin-binding sites located in the N-terminal disordered domain (Kostyukova, Choy & Rapp, 2006).

Tropomyosins (TMs) are a family of rod-shaped, coiled-coil proteins that bind to both grooves of the actin filament (for reviews see (Gunning et al., 2005; Martin & Gunning, 2008)). TMs are encoded by four genes, α, β, γ and δ. Alternative splicing of the genes gives rise to many isoforms, organized by their molecular weight into two classes, short (low molecular weight) and long (high molecular weight) TMs. Distribution of TM isoforms depends on actin isoform localization as well as on the localization of other actin-binding proteins (Gunning et al., 2005; Martin & Gunning, 2008). It is assumed that the microtubule system is also involved in isoform sorting of TMs.

Several TM isoforms are expressed in neurons. Expression depends on the maturity of the neuron and the stage of development. Of all the isoforms, TMBr3 (short α-TM) is the most abundant, but it is expressed in mature neurons only. Other short TMs, TM4 (δ-TM) and TM5NM1 (γ-TM), are expressed early. Expression of TM4 in neurons increases immediately after birth and then declines (Had et al., 1994). Intracellular localization of TM5NM1 changes from axonal to dendritic upon neuronal maturation (Weinberger et al., 1996). Due to differential and strictly regulated patterns of TM expression, it is probable that each isoform is responsible for distinct functions (Gunning et al., 2005; Martin & Gunning, 2008).

Previously, we showed that Tmod-TM interactions are isoform specific (Uversky et al., 2011). Dissociation constants were determined for complexes of synthetic TM and Tmod peptides, which represent binding sites of different Tmod and TM isoforms. We found that binding for short γ- and δ-TMs is very weak in TM-binding site 1 of Tmod1, compared to Tmod2. Changing two residues, Ala 21 and Glu 33 in site 1 of Tmod1 to Lys and Val, the corresponding residues in the Tmod2 sequence, resulted in an increase of γ- and δ-TM binding. We suggested that the function of Tmod isoforms is regulated by their affinity to TM isoforms. In this work, we studied the effects of A21K/E33V mutations in the full-length Tmod1 on the formation of neurites and the binding of short brain TM isoforms. We showed that these mutations caused a decrease in binding of short TMs derived from the α-gene, and that they also caused a 2-fold decrease in the length of neurites formed.

## Materials and Methods

### Plasmid construction and mutagenesis

For transfection experiments and expression in *Escherichia coli*, mouse Tmod2 (accession No. NM_016711) was subcloned into pReceiver-M55 with mCherry tag and into pReceiver-B01 with His-tag (Genecopoeia, Rockville, MD), respectively. The plasmid for mouse Tmod1 (accession No. NM_21883) subcloned into pEGFP-C1 was a generous gift from Dr. Carol Gregorio (Arizona State University) previously used in (Tsukada et al., 2011). The GFP-Tmod1 plasmid was used both for PC12 transfection and as a template for mutagenesis. The pET(His)Tmod1 plasmid (Kostyukova et al., 2000) was used for Tmod1 expression in *E. coli* and as a template for mutagenesis. Site-directed mutagenesis was performed using a QuikChange Site-Directed Mutagenesis Kit (Stratagene, La Jolla, CA). The plasmids were amplified by PCR according to the manufacturer’s instructions with the modification described in (Tsukada et al., 2011) using Pfu Ultra Hotstart DNA polymerase (Agilent) and two complementary sets of oligonucleotides, which contain changed triplets.

To change Ala 21 to Lys in chicken or mouse Tmod1, the sets of oligonucleotides were 5′-GAA GAC AAG ATC CTC GGA AAG CTG ACG GAG GAA GAG CTC-3′ and 5′-GAG CTC TTC CTC CGT CAG CTT TCC GAG GAT CTT GTC TTC-3′; or 5′-GAG GAT GAA ATC CTG GGG AAG CTC ACA GAG GAG GAG C-3′ and 5′-GCT CCT CCT CTG TGA GCT TCC CCA GGA TTT CAT CCT C-3′, respectively.

Then once a single mutation was introduced and confirmed for each plasmid, the singly-mutated plasmid was then used as a template to introduce the second mutation.

To change Glu 33 to Val in chicken or mouse Tmod1, the sets of oligonucleotides were: 5′-CTC AGG AAG TTG GAG AAC GTG CTG GAA GAG CTG GAC-3′ and 5′-GTC CAG CTC TTC CAG CAC GTT CTC CAA CTT CCT GAG-3′, or 5′-CTG AGG ACG CTG GAA AAT GTG CTA GAT GAA CTA GAC-3′ and 5′-GTC TAG TTC A TCT AGC ACA TTT TCC AGC GTC CTC AG-3′, respectively.

After PCR, the original plasmid was digested using DpnI. The digest was used to transform *E. coli* maximum-efficiency DH5α (Invitrogen). Cells were grown in the presence of 100 mg/L carbenicillin for all plasmids except the one for GFP-Tmod1, which was grown in the presence of 50 mg/L kanamycin. After plasmid purification (using Qiagen mini-prep kit), the presence of mutations was confirmed by DNA sequencing. Synthesis of all oligonucleotides was done by Integrated DNA Technologies Inc. (Coralville, Iowa), and DNA sequencing was done by Genewiz (South Plainfield, NJ).

### Tmod purification

Tmod1 (both wild type and mutant) was overexpressed in *E. coli* BL21 (DE3) pLysE while Tmod2 was overexpressed in *E. coli* BL21 (DE3). Auto-inducible ZYP-5052 media (Studier, 2005) containing 100 mg/L carbenicillin (plus 50 mg/L chloramphenicol for *E. coli* BL21 (DE3) pLysE) was inoculated and cells were grown for 12–15 hours at 37 °C. Cells were harvested by centrifugation at 8,000 rpm (Sorvall SLA-3000 Rotor), 4 °C, for 10 min. Pellets were re-suspended in 20 mM Tris-HCL, pH 7.0, 100 mM NaCl, containing a protease inhibitor cocktail (Roche), containing 1 mM pefabloc, and 1 mM tosyl-L-lysine chloromethyl ketone (TLCK). Re-suspended pellets were sonicated for 10 min on ice. The homogenized solution was then centrifuged at 20,000 rpm (Sorvall SA-300 Rotor), 4 °C, for 20 minutes and the supernatant was loaded onto a Superflow Ni-NTA agarose column (Qiagen) equilibrated with 50 mM Na-phosphate, pH 6.8, containing 10 mM imidazole. Once the protein was loaded, the column was washed with 50 mM Na-phosphate, pH 6.8, containing 10 mM imidazole and 5 mM β-mercaptoethanol. Proteins were eluted by a 50–250 mM imidazole gradient in the same buffer. Fractions containing Tmod were combined, dialyzed overnight against 20 mM Tris-HCl, pH 8, containing 1 mM EDTA and 1 mM DTT, and loaded on an anion-exchange column Poros HQ/L (PerSeptive Biosystems) using an FPLC system (Pharmacia). Proteins were eluted by a 12–25% (for Tmod1) or 10-30% (for Tmod2) NaCl gradient. Fractions containing Tmod were combined and dialyzed against 20 mM Tris-HCl, pH 8, containing 1mM EDTA and 1mM DTT. Protein purity was evaluated using SDS-PAGE.

### Peptides

N-acetylated TM peptides (αTm1bzip, γTm1bzip, and δTm1bzip) were synthesized by the Tufts University Core Facility (Boston, MA). Chimeric TM peptides designed for structural and functional studies (Greenfield et al., 2001; Kostyukova, 2007; Meshcheryakov et al., 2011) contain 19 N-terminal residues of short α-, γ-, or δ-TM respectively, encoded by exon 1b. To stabilize the coiled-coil structure, each peptide contained an additional 18 C-terminal residues of the GCN4 leucine Zipper domain. Quality of the synthetic peptides was confirmed using mass spectroscopy; molecular weights of the peptides were the same as the predicted ones.

Isoelectric points of TM peptides were calculated using protparam http://web.expasy.org/protparam/. Concentrations of proteins and peptides were determined both by using the BCA protein assay kit (Pierce) and by measuring their difference spectra in 6 M guanidine-HCl between pH 12.5 and 7.0, using the extinction coefficients of 2357 per tyrosine and 830 per tryptophan at 294 nm as in (Kostyukova, Hitchcock-Degregori & Greenfield, 2007). Standard deviations of concentrations determined by the two methods were 20% for Tmod2 and between 4 and 16% for TM peptides.

### Binding experiments

Binding was detected using native gel electrophoresis in 9% polyacrylamide gels that were polymerized in the presence of 10% glycerol without SDS. To prepare complexes for loading onto gels, stock solutions of 12 µM Tmods were mixed with TM peptides in a 1:2 molar ratio in 20 mM Tris-HCl, pH 8.0, containing 1 mM DTT and 1 mM EDTA. Presence ( ≤ 100 mM) or absence of NaCl did not affect binding results. Sample loading buffer (125 mM Tris-HCl, pH 6.8, 40% glycerol, 0.01% bromophenol blue) was added to the samples to a Tmod final concentration of 7.5 µM. Running buffer for electrophoresis contained 25 mM Tris-HCl and 192 mM glycine. For titration, TM peptides were added at different ratios (up to 3:1) to the stock solution of Tmod2. Samples were analyzed by staining native polyacrylamide gels with Coomassie R-250 and quantified using a ChemiDoc XRS+ with Image Lab Software (BioRad). Density of the complex bands was normalized in order to average results of several titrations.

### Cell culture and imaging

Undifferentiated PC12 cells were grown in DMEM (Invitrogen) supplemented with 5% fetal bovine serum (FBS, Invitrogen) and 10% horse serum (HS, Invitrogen), and were sub-cultured every three days. For neuronal differentiation, PC12 cells were grown on 100 µg/ml poly-D-lysine–coated 8-well plates or 35-mm culture dishes (ibidi LLC) in DMEM supplemented with 0.5% FBS, 1% HS, and 100 ng/ml nerve growth factor (NGF, invitrogen). PC12 cells were transfected with GFP-Tmod1 WT, GFP-Tmod1 A21K/E33V, mChFP-Tmod2, or GFP-actin using lipofectamin 2000 (Invitrogen) according to the manufacturer’s instructions. Twenty-four hours after transfection, neuronal differentiation was induced and neurite outgrowth was monitored on day 3 and day 6 on a laser scanning confocal microscope LSM710 (Zeiss) with a 63x oil immersion lens (Zeiss). Images were acquired with Zen software (Zeiss) and the number and length of neurites were analyzed in Imaris software (Bitplane). Statistical analysis was performed by One-way ANOVA.

## Results

### A21K and E33V mutations in Tmod1 cause decrease in neurite length

Mutagenesis of Ala21 to Lys and Glu33 to Val in the Tmod1 fragment, Tmod1s1, increased the affinity of this fragment for TM peptides (Uversky et al., 2011). Tmod1s1 corresponded to the TM-binding site 1 of Tmod1 (res. 1-38). TM chimeric peptides, γTM1bzip and δTM1bzip, contained the N-terminal sequences of short TM isoforms TM5NM1 and TM4, respectively. To study the effects of this changed affinity on neurite formation, these mutations were introduced in full-length GFP-Tmod1. The mutant was expressed in PC12 cells, a model system for neuronal differentiation (Greene & Tischler, 1976) and functional assessment of neurite outgrowth (Guillaud et al., 1998), to test its effect on neurite formation and extension.

**Figure 1:**
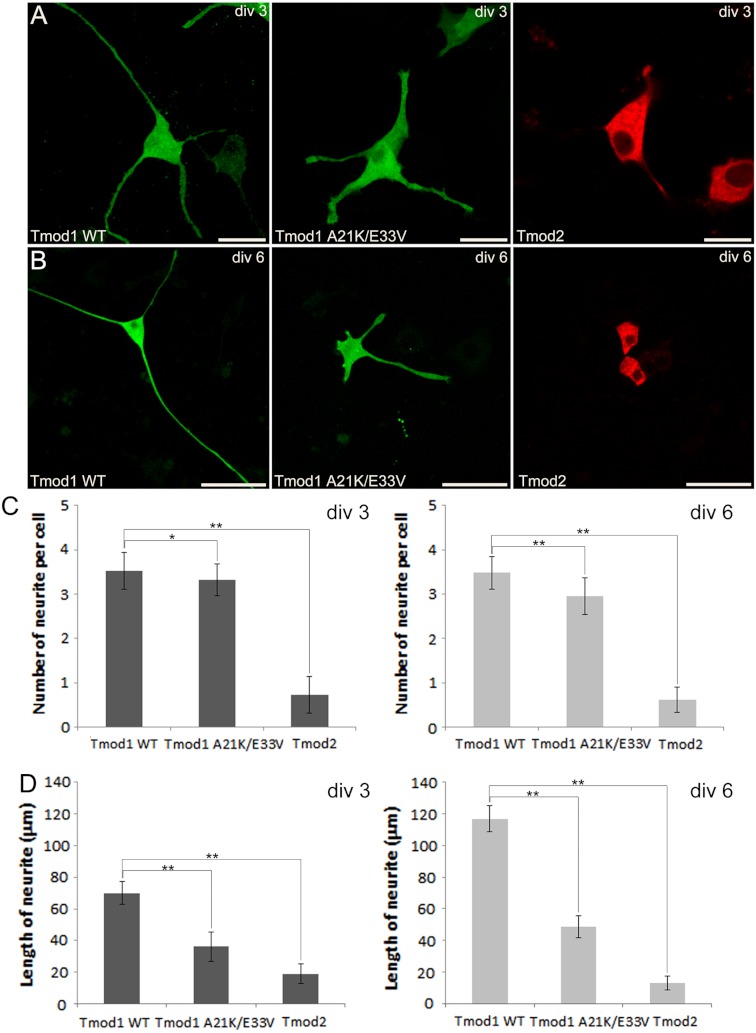
Confocal imaging illustrating the effect of Tmod expression on PC12 neuronal differentiation. (A) PC12 cells over-expressing GFP-Tmod1 wild-type, GFP-Tmod1 mutant, and mChFP-Tmod2 after three days of NGF-induced neuronal differentiation. Bar = 20 µm. (B) PC12 cells over-expressing GFP-Tmod1 wild-type, GFP-Tmod1 mutant and mChFP-Tmod2 after six days of NGF-induced neuronal differentiation. Bar = 50 µm. (C) Bar graphs showing the number of neurites per cell and (D) the average length of neurites in PC12 cells over-expressing GFP-Tmod1 WT (*n* = 55), GFP-Tmod1 A21K/E33V (*n* = 48) and mChFP-Tmod2 (*n* = 45) three days (dark grey bars) and six days (light grey bars) after NGF-induced neuronal differentiation. Error bars in C) and D) represent sd (*: *p* < 0.01 and **: *p* < 0.0005; One-way ANOVA).

**Figure 2:**
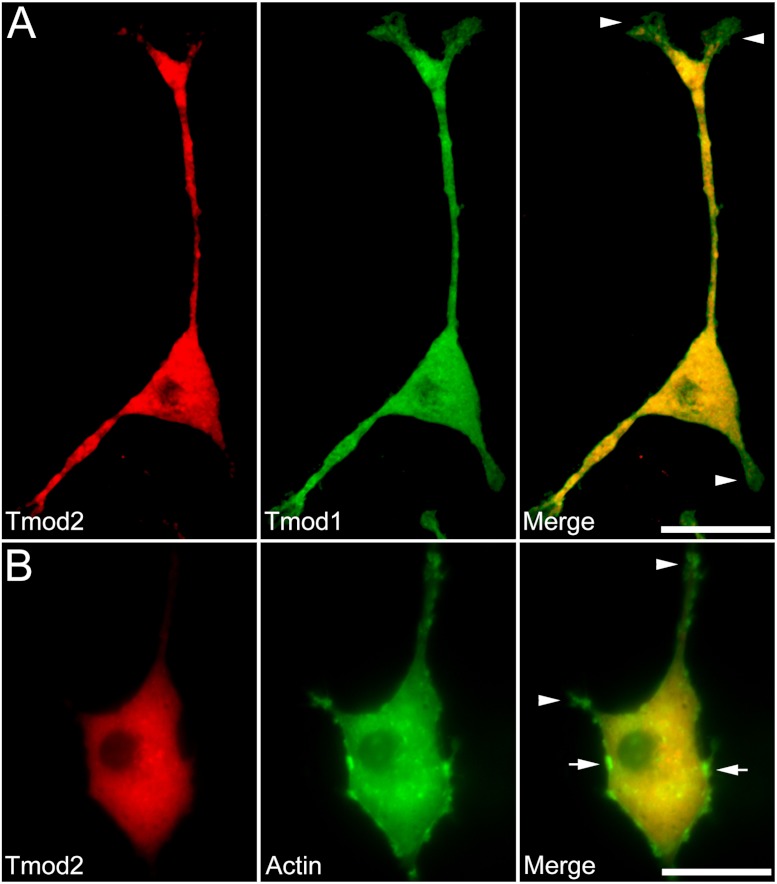
Localization and distribution of Tmod2 in PC12 cells. A) Co-expression of mChFP-Tmod2 and GFP-Tmod1 in PC12 cells after 3 days of NGF-induced neuronal differentiation. Tmod2 does not co-localize with Tmod1 in the growth cones (arrowheads). Bar = 20 µm. B) Co-expression of mChFP-Tmod2 and GFP-actin in PC12 cells after 3 days of NGF-induced neuronal differentiation. Tmod2 does not co-localize on actin filaments in the tip of the neurites (arrowheads) or membrane ruffles (arrow). Bar = 20 µm.

We first checked expression of GFP-Tmod1 and mChFP-Tmod2 in PC12 cells. Both GFP-Tmod1 and mChFP-Tmod2 were successfully expressed and localized in both the cell body and neuronal extensions in differentiated PC12 cells (Fig. 1A); however, mChFP-Tmod2 appeared be more restricted to the shaft of the neurites, while GFP-Tmod1 localized in the shaft and at the very end of the growth cones (Fig. 2A).

We next observed the effect of GFP-Tmod1 and mChFP-Tmod2 overexpression on neurite formation and outgrowth of PC12 cells, three and six days after NGF induction. Overexpression of GFP-Tmod1 did not affect neuronal differentiation when compared with overexpression of GFP alone (data not shown), but overexpression of mChFP-Tmod2 impaired neuronal differentiation of PC12 cells (Figs. 1A and 1B). PC12 cells expressing GFP-Tmod1 showed proper neurite outgrowth after three and six days of NGF-induced neuronal differentiation. On the other hand, PC12 cells expressing mChFP-Tmod2 showed a significant reduction in the number (4-fold) and the length (3 to 10-fold) of neurites (Figs. 1C and 1D).

Additionally, we co-expressed GFP-Tmod1 and mChFP-Tmod1 in PC12 cells and checked their effect on neuronal differentiation. Interestingly, co-expression of Tmod1 and Tmod2 did not impair NGF-induced neuronal differentiation after three days (Fig. 2A). The number (3.1 ± 0.6, *n* = 20) and length (61.3 ± 8.3 µm, *n* = 20) of neurites were similar to those observed in Tmod1 over-expressing cells (Figs. 1C and 1D). Tmod2 did not localize with actin filaments in the tips of neurites of PC12 cells over-expressing mChFP-Tmod2 and GFP-actin (Fig. 2B).

We further checked the effect of A21K and E33V mutations in GFP-Tmod1 on PC12 cell’s neuronal differentiation. PC12 cells overexpressing GFP-Tmod1[A21K/E33V] did not exhibit a significant decrease in the number of neurites per cell; however, the average length of neurites decreased approximately 2 to 3-fold (Figs. 1A–1C). Interestingly, GFP-Tmod1[A21K/E33V] shared a similar localization pattern with mChFP-Tmod2 (data not shown).

### Effect of the mutations in full-length Tmod1 on binding TM isoforms

Experiments that demonstrated increased Tmod1 binding to short non-muscle TM isoforms were performed using synthetic TM and Tmod peptides (Uversky et al., 2011). Residues 7–14 of short TMs form the binding site for Tmod (Vera et al., 2000). Therefore the data obtained for TM peptides, which contain 19 N-terminal residues, may be extrapolated to full-length TMs. Tmods contain two TM-binding sites; changes in one site may or may not affect TM-binding properties of the full-length molecule.

**Figure 3:**
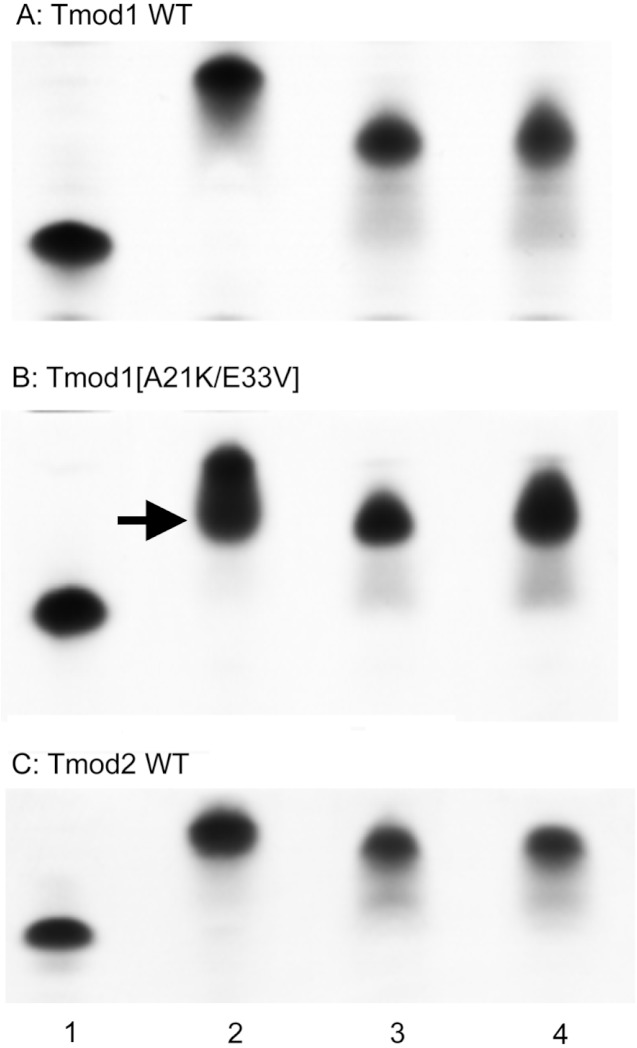
Complex formation between Tmods and TM peptides detected by shift in native gel-electrophoresis. Tmod and TM peptides were mixed at 1:1 ratio. Tmod (lane 1), Tmod and αTM1bzip (lane 2), Tmod and γTM1bzip (lane 3), Tmod and δTM1bzip (lane 4). TM peptides cannot be seen in gels because of high isoelectric points. Arrow indicates the additional complex band.

To explore the effect of the changes on full-length Tmod1, A21K and E33V mutations were introduced into full-length, His-tagged Tmod1. TM-binding properties of purified mutant Tmod1[A21K/E33V] and wild-type Tmod1 and Tmod2 were analyzed using native gel-electrophoresis (Fig. 3). Three TM peptides, αTM1bzip, γTM1bzip and δTM1bzip, were used in this experiment. All of them contained TM N-terminal sequences encoded by exon 1b of α, γ, and δ-genes, respectively. Tmods alone and mixed with TM peptides were loaded on native gels. TM peptides cannot be seen on native gels; they are positively charged and do not enter a gel unless they are in a complex with Tmod (isoelectric points of αTM1bzip, γTM1bzip and δTM1bzip are 9.98, 9.82, and 9.82, respectively). Binding positively charged TM peptides decreased Tmod mobility in gels; therefore, a shift of bands corresponding to the formed complexes was observed.

**Figure 4:**
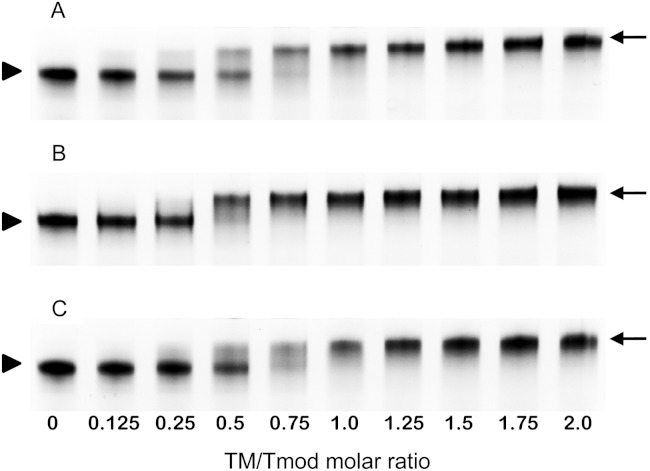
Titration of Tmod2 by TM peptides αTM1bzip (A), γTM1bzip (B) and δTM1bzip (C). Arrows indicate complexes, arrowheads indicate Tmods.

Compact bands were formed for the mixtures of both Tmod1 and Tmod2 with αTM1bzip, (Fig. 3, lane 2) indicating formation of a stable complex. Tmod1 has been shown to bind an αTM1bzip peptide at both sites (Kostyukova, Choy & Rapp, 2006). Although Tmod1 also has two binding sites for γTM1bzip, formation of a complex with only one γTM1bzip peptide bound to site 2 can be detected using native gel-electrophoresis (Kostyukova, Hitchcock-Degregori & Greenfield, 2007). Binding one peptide instead of two resulted in higher electrophoretic mobility of the Tmod1/γTM1bzip band on the gel, and resulted from the weak affinity of γTM1bzip to site 1. The similar position of the band for the Tmod1/δTM1bzip complex, indicated that only one δTM1bzip peptide was bound to Tmod1 under these conditions (Fig. 3A, lane 4). A smear, which appeared under the Tmod1/γTM1bzip and Tmod1/δTM1bzip bands, can be explained by complex dissociation during electrophoresis, indicating weaker affinities of Tmod1 with these TM isoforms (Fig. 4A, lanes 3–4).

Mutations A21K and E33V were designed based on previous data (Uversky et al., 2011) to enhance interaction of Tmod1 with short γ- and δ-TMs in site 1. With these mutations, we expected to see a band with the mobility of the band formed by Tmod1 and αTM1bzip. However, we saw no change of mobility for the complexes of Tmod1[A21K/E33V] with γTM1bzip and δTM1bzip (Fig. 4B, lanes 3, 4). Unexpectedly, rather than a single band, for the complex of Tmod1[A21K/E33V] with αTM1bzip, an additional lower band appeared (Fig. 3B, lane 2). The usual band representing a complex of Tmod1[A21K/E33V] with two αTM1bzip was also observed. The increased mobility of the additional band indicated formation of a complex with one TM peptide. Therefore, these mutations decreased the affinity of Tmod1 for the αTM1bzip in site 1, but did not affect the formation of the complex of Tmod1 with the two other TM peptides at least that can be detected by native gel-electrophoresis.

The mobilities of the Tmod2/γTM1bzip and Tmod2/δTM1bzip bands were slightly greater than the mobility of Tmod2/αTM1bzip (Fig. 3C). The difference may be explained either by the slightly higher isoelectric point of αTM1bzip or by binding only one peptide.

### Titration of Tmod2 by TM peptides

**Figure 5:**
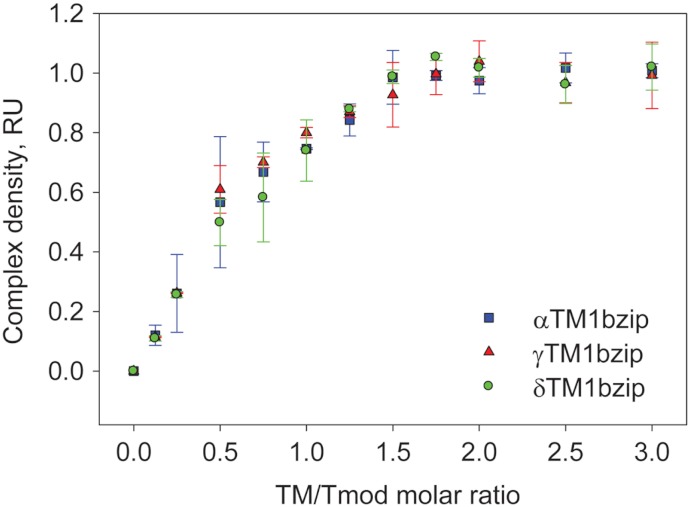
Density dependence of the complex bands on the amount of TM peptide added (TM/Tmod molar ratio). Error bars show standard deviation (*n* = 3).

To find out how many TM molecules bind to Tmod2, Tmod2 was titrated with αTM1bzip, γTM1bzip, and δTM1bzip (Fig. 4). In all cases, the Tmod2 band disappeared completely at a 1:1 molar TM/Tmod ratio, indicating that at this ratio all Tmod2 molecules were in a complex with one TM peptide. Complex formation between Tmod2 and αTM1bzip was different than with the two other peptides. The intensity of the Tmod2/αTM1bzip band continued to increase gradually, reaching maximum at a 2:1 ratio. It was more difficult to understand if the intensity of complex bands for Tmod2/γTM1bzip and Tmod2/δTM1bzip also continued to increase after disappearance of Tmod2 band. To clarify this, the change of the complexes in the mixture was monitored by scanning and quantifying the complex bands in native gels. The normalized density of the complexes is shown in Fig. 5 as a function of the TM/Tmod molar ratio. All curves demonstrate saturation at a 2:1 ratio. This result may be explained if binding of Tmod2 to all peptides was not cooperative and all Tmod2 molecules bind one TM peptide first, in a site that has a higher affinity; then a second peptide starts to bind. The change in the position of the complex band also confirms this explanation. Mobility of the complex band at a 1:1 ratio is slightly higher than at a 2:1 ratio (Fig. 4).

## Discussion

TM5NM1, TM4 and TMBr3 are short TM isoforms specific to neurons. Location and development profiles of these isoforms were studied in rats using isoform-specific antibodies (Had et al., 1994; Weinberger et al., 1996; Schevzov et al., 2005; Martin & Gunning, 2008). There is a low level of TMBr3 at birth with an increase after day 10 and the maximum level at days 21-35. Levels of TM4 are high at birth and reach a maximum at day 10, followed by decreasing levels. It was shown that in growing embryonic neurons, TM4 was concentrated in the cell body and the growth cones located at the distal ends of neurites. In embryonic neurons, TM5NM1 is localized in the growth cones, but cannot be found in cell bodies. In adult neurons, both TM5NM1 and TM4 localize in the cell body while TMBr3 was found in the axon and presynaptic area.

Tmod2 was found in the brain along with Tmod1 as a TMBr3-binding protein and was identified as a new Tmod isoform specific for neurons (Watakabe, Kobayashi & Helfman, 1996). Tmod2 is detected at embryonic day 14 and reaches adult levels before birth. Watakabe et al. also tested binding of Tmod1 and Tmod2 to several TM isoforms: TM5a, TM5NM1, TMBr3 and TM4. They found strong binding of both Tmod1 and Tmod2 to TM5a, TMBr3 and TM5NM1. Weak binding to TM4 was detected for Tmod1 and no TM4 binding was detected for Tmod2. However, we showed that Tmod2 binds TM4. This conclusion is supported by our results with δTM1bzip and the drastic increase in Tmod2’s ability to cap actin filaments in the presence of full-length TM4 (data not shown). Therefore, binding to all these isoforms should be important for Tmod2 function in neurons.

Originally the chimeric peptide αTM1bzip was designed to study the structure and function of TM5a/5b, the short non-muscle α-TMs (Greenfield et al., 2001; Kostyukova, 2007; Meshcheryakov et al., 2011). TM5a was reported in brain tissue (Schevzov et al., 2005) with a polyclonal a/9d antibody that cross-reacted with TM5NM1. However, the monoclonal a/9d antibody, which did not cross-react with TM5NM1, did not detect TM5a (Schevzov et al., 2011); therefore, most likely this TM isoform is not specific for neurons. Two other short TM isoforms, TMBr2 and TMBr3, result from splicing of the α-gene and share the same N-terminal region encoded by exons 1b, 3, 4 and 5 (Gunning et al., 2005). Therefore, the Tmod-binding properties of αTM1bzip, which contains the sequence encoded by exon 1b, may be attributed to all these isoforms.

Our present results regarding the localization of overexpressed Tmod1 and Tmod2 are in agreement with the literature investigating localization of Tmod1 and Tmod2 in cultured neurons (Fath et al., 2011). Fath and coauthors showed that Tmod2 was localized in the cell body and central domain of the growth cone and most of it was diffuse and not associated with actin filaments during early neuritogenesis. Unlike Tmod2, most of Tmod1 was co-localized with actin filament bundles in lamellipodia and growth cones.

Knockdown of Tmod2 in N2a neuroblastoma cells increased the percentage of cells with neurites (no change in number of neurites) and the length of neurites was increased 2-fold (Fath et al., 2011). Knock-down of Tmod1 had no effect on the percentage of cells with neurites, but increased the number of neurites per cell and slightly decreased their lengths. It was concluded that Tmod2 negatively regulates neurite formation and extension; and the decreases in neurite formation and elongation observed in Tmod2 over-expressing PC12 cells presented here, strongly confirm and support the above hypothesis.

Fath and co-authors (Fath et al., 2011) suggested that Tmod2’s ability to bind G-actin (Fischer et al., 2006; Yamashiro et al., 2010) might explain the inhibition of neurite growth, because Tmod2 sequesters free actin monomers in the cytoplasm. However, in our experiments, co-expression of Tmod1 and Tmod2 did not change the number and length of neurites; therefore, the sequestering activity of Tmod2 did not affect neurite growth.

The slight decrease in neurite number and the 2-fold decrease of neurite length that resulted from the A21K/E33V mutation in TM-binding site 1 of Tmod1, indicate that the difference in TM-binding abilities is important for the different functions of Tmod isoforms. Unexpectedly, the A21K/E33V mutation in Tmod1, that was supposed to increase binding to TM5NM1 and TM4 in site 1 (Uversky et al., 2011), also decreased binding of αTM1bzip and therefore of a short α-TM, TMBr3. However, actin filaments in the growth cone are associated with TM5NM1 and TM4 while TMBr3 is expressed only in adult neurons. We assume the most likely it is the change in binding TM5NM1 and TM4 that caused the decrease in neurite length.

Our present data on the over-expression of Tmod1 WT, Tmod1[A21K/E33V] and Tmod2 in PC12 cells clearly argue that Tmod1 is involved in neuronal differentiation for proper neurite formation and outgrowth, and that Tmod2 inhibits such neuronal differentiation. In fact, it is possible that an adequate balance between Tmod1 and Tmod2 levels may regulate actin polymerization in the growth cone during neuronal differentiation in order to promote neurite extension. Tmod1 may be required at the tip of growth cones whereas Tmod2 may be necessary within the shaft of the neurite. Mutations in the TM binding site of Tmod1 significantly impair neurite outgrowth, suggesting that the integrity of this binding domain is critical for the proper function of Tmod1 during neuronal differentiation.
